# Whole-Genome sequencing of Chikungunya Virus (CHIKV) from Pakistan: Detection of the East/Central/South African (ECSA) genotype during the 2024 outbreak in Mansehra

**DOI:** 10.1371/journal.pone.0329856

**Published:** 2025-08-26

**Authors:** Massab Umair, Muhammad Yasir, Zunera Jamal, Rabia Hakim, Syed Yawar Saeed, Muhammad Salman, Azhar Iqbal, Muhammad Qasim Khan, Faisal Khanzada, Yusra Javed

**Affiliations:** 1 Department of Virology, National Institute of Health, Chak Shahzad, Islamabad, Pakistan; 2 District Health Office Manshera 85VW+WHW, Mansehra, Khyber Pakhtunkhwa, Pakistan; 3 Health Services Academy, Chak Shahzad, Islamabad, Pakistan; Kwame Nkrumah University of Science and Technology, GHANA

## Abstract

The 2024 chikungunya virus (CHIKV) outbreak in Mansehra, Khyber Pakhtunkhwa, Pakistan, marked a significant public health event, providing a unique opportunity to investigate the genomic diversity and evolutionary dynamics of circulating strains. Using metagenomic next-generation sequencing (mNGS), we analyzed serum samples from patients presenting with acute febrile illness and joint pain, identifying 16 CHIKV-positive cases, six of which yielded near-complete genomes. Phylogenetic analysis revealed that all isolates belonged to the East/Central/South African (ECSA) genotype, closely related to strains from India (2020–2024) and China (2017). Notably, this study represents the first comprehensive whole-genome sequencing of CHIKV in Pakistan, uncovering unique mutations in structural (E1: I28V, V290I; E2: Y39H, D54E) and non-structural proteins (NSP1: I167V, M376T; NSP4: G27R, P52S, I403V), suggesting potential viral adaptations to local environmental and vector conditions. The absence of the E1-A226V mutation, associated with enhanced transmission by *Aedes albopictus*, highlights the need for continued genomic surveillance to monitor emerging variants. Additionally, the detection of a GB virus-C co-infection in one case underscores the utility of mNGS in identifying co-circulating pathogens. This study provides critical insights into the genomic landscape of CHIKV in Pakistan, emphasizing the importance of enhanced surveillance, diagnostics, and vector control strategies to mitigate future outbreaks. The findings underscore the necessity of regional collaboration and genomic monitoring to address the evolving threat of CHIKV in South Asia.

## Introduction

Chikungunya virus (CHIKV) is a re-emerging mosquito-borne *Alphavirus* in the *Togaviridae* family, transmitted to humans by *Aedes* mosquitoes *(A. aegypti and A. albopictus*) [1]. It typically causes mild, self-limiting symptoms, but poses greater risks to vulnerable populations, such as infants, the elderly, and individuals with underlying health conditions [2,3]. The symptom overlap between CHIKV and other arboviruses, such as Dengue and Zika, underscores the critical need for accurate laboratory diagnostics to avoid misdiagnosis [1].

CHIKV has a small (~11.8 kb), enveloped, single-stranded positive-sense RNA genome with two open reading frames (ORFs). The 5′ ORF encodes non-structural polyproteins (nsP1-nsP4) essential for replication and host adaptation, while the 3′ ORF encodes structural proteins (C, E1-3, 6K/TF) responsible for receptor binding, membrane fusion, and virus assembly [4,5]. Phylogenetic studies classify CHIKV into three major genotypes: Asian (AS), West African (WA), and East/Central/South African (ECSA) [6]. The ECSA genotype consists of two clades, ECSA1 and ECSA2, with sequences from the Republic of Congo, Cameroon, Gabon, and the Central African Republic [7]. The Indian Ocean Lineage (IOL), a sub-lineage of ECSA, emerged during the 2005–2006 epidemic due to the E1-A226V mutation [8]. Additional mutations (E2-I211T, E1-T98A, E2-L210Q) further enhanced IOL’s affinity for the *Aedes albopictus* vector, increasing CHIKV transmission. These genetic changes underscore the virus’s potential for reemergence and sustained transmission [9].

CHIKV was first isolated in 1952 in Tanganyika (now Tanzania) and remained largely confined to Asia and Africa until the early 2000s, when outbreaks began to occur more frequently [10]. In 2005, CHIKV reemerged, spreading across Southeast Asia, the Indian Ocean islands, and India, with over 1 million cases (likely underreported) [1]. In 2006, Sri Lanka reported fewer than 100,000 cases, while Thailand saw 266,000 cases between 2008 and 2009. The Americas were heavily impacted after the first locally acquired cases in the Caribbean in late 2013, leading to over 1 million suspected cases by 2014. Brazil experienced a major outbreak from 2014 to 2015, and India saw a significant surge in 2016–2017, reporting over 26,000 cases. Outbreaks continued in Southeast Asia, with notable cases in Thailand, Indonesia, and Malaysia in 2019. By 2022, 64 cases were reported across 26 EU/EEA countries, most originating from Asia [11–19]. In 2023, over 460,000 cases and 350 deaths occurred, mainly in South and Central America [20]. In 2024, the global total reached approximately 480,000 cases and 190 deaths, with Brazil reporting the highest number (403,726 cases), followed by India (69,544 cases) and Pakistan (4,964 cases) [21].

Pakistan, with a population exceeding 240 million [22], has a diverse climate, from hot, dry conditions in the plains to cooler temperatures in the northern regions [23]. While dengue is endemic in Pakistan, with frequent outbreaks, CHIKV cases have been rare, despite the widespread presence of mosquito vectors such as *Aedes aegypti and Aedes albopictus* [11,24]. Serological evidence of CHIKV in Pakistan was first reported in rodents in 1983 [25]. During the 2011 dengue outbreak in Lahore, some patients tested positive for CHIKV antibodies, but no further cases were recorded until December 2016, when a confirmed case in Karachi led to outbreaks in Sindh and Khyber Pakhtunkhwa (KPK), totaling 2,582 confirmed cases [26]. By 2017, suspected and confirmed cases rose to 8,387 [27]. Phylogenetic studies confirmed these strains belonged to the Indian Ocean Lineage with the ECSA genotype [24]. From 2019 to 2023, sporadic cases of CHIKV were reported primarily in Karachi, Sindh. However, a notable resurgence in CHIKV cases has been observed in 2024, particularly in Karachi. In September 2024, a localized outbreak was reported in Mansehra district in KP, Pakistan, raising concerns about the geographic expansion of the virus.

Although Pakistan’s public health laboratory system has recently initiated CHIKV testing, surveillance remains limited, and genomic data on circulating strains is scarce. Previous studies have provided partial sequence data of the virus [24,28–30], however, comprehensive insights into the genetic diversity of CHIKV in Pakistan are lacking due to inadequate genomic surveillance and sequencing capacity. To address this gap, the current study identifies the CHIKV genotype and explores the genomic diversity of strains circulating in the Mansehra district of KP using metagenomic next-generation sequencing (mNGS).

## Methodology

### Sample collection

This study was conducted as a prospective genomic surveillance investigation utilizing retrospectively collected diagnostic samples. Serum samples were collected between September and November 2024 from patients in Mansehra district, Khyber Pakhtunkhwa, Pakistan, who presented with acute febrile illness and joint pain amid a suspected CHIKV outbreak. These samples were referred to the Department of Virology, National Institute of Health (NIH), Islamabad, for diagnostic testing and genomic surveillance purposes. Alongside sample collection, demographic and clinical data were also gathered.

For genomic surveillance, metagenomic next-generation sequencing (mNGS) was conducted on residual anonymized clinical diagnostic samples. The Institutional Review Board (IRB) of NIH approved the processing of these samples for genomic surveillance and granted a waiver for informed consent to facilitate knowledge building, surveillance, and outbreak response efforts. Data was accessed on 07-01-2025.

All samples and associated metadata were anonymized prior to analysis. No personally identifiable information (PII) was accessed or used in this study.

### Nucleic acid extraction and real time PCR

Viral RNA of all collected specimens was extracted using the QIAamp viral RNA mini kit (Qiagen, Germany) according to manufacturer instructions. All samples were tested for Zika, Dengue and Chikungunya virus according to manufacturer’s instructions by using Vircell Trioplex real‐time RT‐PCR (ZIKV/DENV/CHIKV) kit (Granada, Spain) [31]. The assay was used to confirm the presence of CHIKV in 14 samples from Mansehra district, which tested positive for chikungunya virus by RT-PCR.

### Next Generation Sequencing (NGS)

In this study, we selected CHIKV samples with Ct values ≤32 for shotgun metagenomics sequencing. These samples were prepared for unbiased paired-end sequencing using the NEBNext Ultra II Directional RNA Library Prep Kit (New England Biolabs) according to the manufacturer’s instructions. For library barcoding, we used NEBNext Multiplex Oligos for Illumina Dual Index Primers Set 1 (New England Biolabs). Library concentrations were measured using a Qubit 4.0 fluorometer and Qubit dsDNA HS assay kit (Invitrogen), while library sizes were evaluated with an Agilent Bioanalyzer and the DNA 1000 Kit (Agilent Technologies). Each library was diluted to 4 nM, combined in equal proportions, denatured with 0.2 N sodium hydroxide, and further diluted to a final concentration of 10 pM. Sequencing was carried out on an Illumina MiSeq system using the MiSeq Reagent Kit v2 (300 cycles).

### NGS data analysis

Raw NGS reads were initially assessed for quality using the FastQC v0.12.0 program [32]. Low-quality reads and adapter sequences were trimmed by Trimmomatic (v.40) with the parameters ILLUMINACLIP:adapters-PE.fa:2:30:10, LEADING:3, TRAILING:3, SLIDING WINDOW: 4:30, MINLEN:50 [33]. The filtered reads were assembled into contigs with SPAdes v4.0.0 following the default settings [34] and then aligned to the NCBI Non-redundant (NR) database to identify the most similar genome using the Burrows-Wheeler Aligner (BWA) v0.7.18 [35]. PCR duplicates were subsequently removed from the aligned reads using the Picard MarkDuplicates tool (https://broadinstitute.github.io/picard/). Finally, consensus genomes were generated in Geneious Prime v2024.0 with following parameters (threshold = 0%, Assign Quality = total, minimum coverage >10) [36]. Genotyping of these samples was performed by using the Genome Detective (CHIKUNGUNYA VIRUS TYPING TOOL (v.3.7.1) (https://www.genomedetective.com/app/typingtool/chikungunya/). The resultants sequences have been submitted with accession numbers: CHIKV (NCBI# PV054360 to PV054365).

### Phylogenetic analysis

Whole-genome sequences of selected high-coverage CHIKV samples obtained in this study were subjected to the BLAST search tool to identify closely related sequences for phylogenetic analysis. The highest-matching sequences were retrieved from NCBI GenBank (http://www.ncbi.nlm.nih.gov/) and GISAID (https://gisaid.org/) as of November 11, 2024. These sequences were then further filtered based on near-complete sequence length, fewer than 2% ambiguous bases (N’s), and a complete collection date. To ensure diversity, sequences that matched identically or differed by only a single nucleotide were excluded from further analysis. Then, the MAFFT aligner (CLI-based, v.7) was employed for multiple sequence alignment. The best-fit substitution model was selected using the ModelFinder within IQ-TREE2 (v2.3.6), resulting in the choice of the Generalized Time Reversible model (GTR + G) [37]. This model was then applied to perform a scaled, unrooted phylogenetic analysis based on the maximum-likelihood approach with 1,000 bootstrap replicates for branch support. The resulting phylogenetic tree was constructed and visualized using MEGA11 [38].

### Mutation analysis

To perform mutation analysis, the whole-genome sequences were initially trimmed based on the open reading frames (ORFs) of the viral genes, including structural proteins (E1, E2, E3) and nonstructural proteins (NSP1, NSP2, NSP3, and NSP4). The trimmed sequences were aligned using ClustalW and translated into standard amino acid sequences with MEGA11. The deduced amino acid sequences were further aligned using MAFFT and compared with previous CHIKV sequences from Pakistan (2016–2020 and 2022) and the CHIKV reference genome (GenBank accession no. MN630017) in MEGA11. Mutation positions were identified and annotated relative to the reference genome.

## Results

Out of the total samples (n = 84) referred to the Department of Virology at the NIH, Islamabad, for routine diagnostic testing, fourteen samples (n = 14) from Manshera district were confirmed positive for CHIKV using the Vircell Triplex real-time RT-PCR assay. Common presenting symptoms of CHIKV positive patients were fever (100%), joint pain (100%), myalgia and nausea (88%). A subset (n = 09) CHIKV-positive samples (Ct value ≤32) from Manhsera district was selected for metagenomic next-generation sequencing. Of these, six samples yielded near-complete genomes with over 99.30% coverage, while one sample showed only 40.07% coverage, and two samples yielded low-quality data with genome coverage below 20% and were excluded from downstream analysis. Genotyping of the successfully sequenced samples revealed the East/Central/South African (II-ECSA) genotype. Notably, one CHIKV sample (PV054362) was co-infected with GB virus C (GBV-C), with 94.87% coverage (**Table 1).**

**Table 1:** Demographic, clinical, and genomic characteristics of CHIKV strains from the 2024 outbreak in Khyber Pakhtunkhwa, Pakistan.

Sr No.	ID	District	DOC	Ct value	Genotype	Genome Coverage	Co-infection	Accession ID
1	CHIKV_2024_NIH_2	Mansehra	9/3/2024	18	ECSA	40.07%	-	EPI_ISL_19429567
2	CHIKV_2024_NIH_6		9/6/2024	19	ECSA	99.86%	-	PV054360
3	CHIKV_2024_NIH_7		9/8/2024	26	ECSA	99.71%	-	PV054361
4	CHIKV_2024_NIH_8		9/6/2024	17	ECSA	99.88%	GB Virus C (94.87%)	PV054362
5	CHIKV_2024_NIH_10		9/8/2024	14	ECSA	99.93%	-	PV054363
6	CHIKV_2024_NIH_14		9/5/2024	26	ECSA	99.30%	-	PV054364
7	CHIKV_2024_NIH_15		9/5/2024	26	ECSA	99.33%	-	PV054365

### Phylogenetic analysis

**Fig 1:**
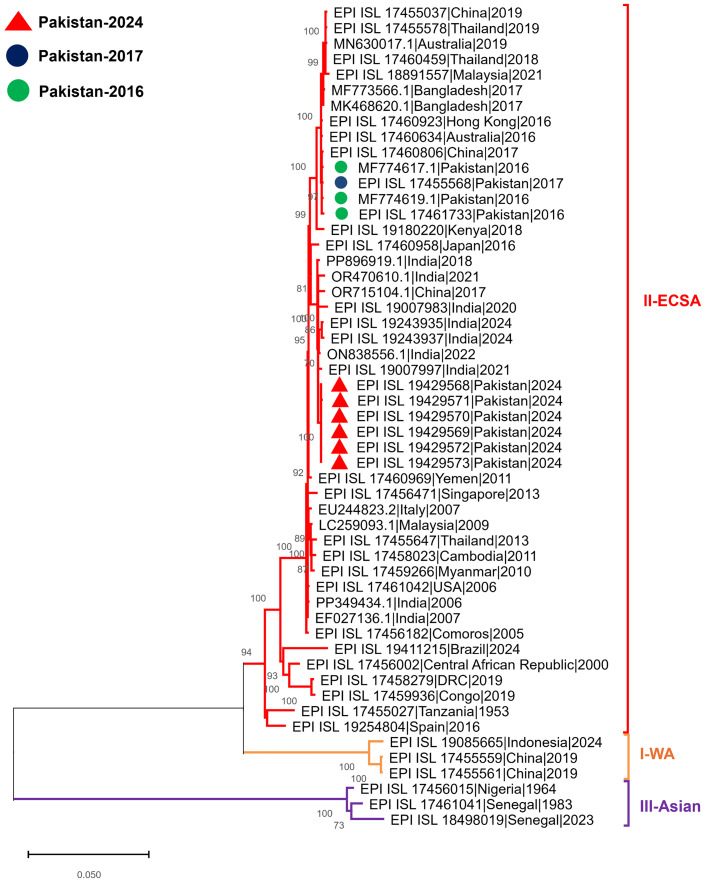
Unrooted Phylogenetic trees of the CHIKV. Study sequences are highlighted in red triangles for 2024, while previous sequences from Pakistan are color-coded as follows: Blue triangle for 2017 and green triangle for 2016. The tree was using the maximum likelihood method with 1,000 bootstrap replicates in MEGA-11 software. The Genotypes are represented by colored dashed lines.

Phylogenetic analysis of six high-coverage study samples (>99.30%) revealed that the CHIKV strains clustered within the ECSA clade, closely related to strains from India (Maharashtra, 2020–2024). Nucleotide identity with these Indian strains ranged from 99.50–99.73% for 2024 strains, 99.48–99.77% for 2021 strains, and 99.47–99.50% for 2020 strains. The study strains also showed high nucleotide homology with strains from Kenya (2018, > 98.72%), China (2017, > 99.56%), and Japan (2016, > 99.18%). Additionally, these strains exhibited 98.91–99.00% nucleotide similarity with previously reported CHIKV strains from Pakistan (2016–2017). Furthermore, the six Pakistani CHIKV sequences demonstrated high nucleotide (~99.9%) homology among themselves (**Fig 1).**

### Mutation analysis

The CHIKV study sequences were compared with previous CHIKV sequences from Pakistan using a reference sequence (GenBank: MN630017.1). This comparison revealed the absence of the known E1-A226V mutation. However, six unique mutations were identified in the structural region: E1 (I28V, V290I), E2 (Y39H, D54E), and E3 (T16M) (**Table 2).** Additionally, eleven mutations were found in non-structural proteins: NSP1 (I167V, M376T), NSP2 (Y39H, D54E), NSP3 (I162V, T373I, V428A), and NSP4 (G27R, P52S, I403V) (**Table 3).** The E2 mutation V386A, located in the transmembrane region, showed similarity to older Pakistani sequences. Notably, the non-structural region exhibited a higher number of unique mutations than the structural region.

**Table 2:** Amino acid variation in the structural genes of CHIKV study isolates in comparison with the reference strain and previously reported sequences from Pakistan.

Proteins	E1		E2				E3
Positions	28	290	131	316	341	344	16
**Thail|MN630017.1| 2019**	I	V	S	M	I	I	T
**Pak|WIV|EPI_ISL_17455568|2017**	*	*	G	*	*	*	*
**Pak|MG516711.1|2018**	*	*	G	*	*	*	*
**Pak|AXB88096.1|2017**	*	*	G	*	*	*	*
**Pak|AXB88094.1|2018**	*	*	G	*	*	*	*
**Pak|AWX90874.1|2018**	*	*	G	*	*	*	*
**Pak|ASY01966.1|2017**	*	*	G	*	*	*	*
**Pak|MF740881.1||2016**	*	*	G	*	*	*	*
**Pak|UYI58226.1| 2018**	*	*	G	*	*	*	*
**Pak|MH548369.1|2018**	*	*	G	*	*	*	*
**Pak|MN42500.1|E1|2020**	*	*	–	–	–	–	–
**Pak|URX6544.1|E1|2022**	*	*	–	–	–	–	–
**Pak|MZ726865.1|E2||2017**	–	–	G	*	*	*	–
**Pak|MZ726886.1|E3|2022**	–	–	–	–	–	–	*
**Pak|UYI58270.1|E3|2017**	–	–	–	–	–	–	*
**Pak|NIH|19429568|2024**	V	I	G	I	V	V	M
**Pak|NIH|19429569|2024**	V	I	G	I	V	V	M
**Pak|NIH|19429570|2024**	V	I	G	I	V	V	M
**Pak|NIH|19429571|2024**	V	I	G	I	V	V	M
**Pak|NIH|19429572|2024**	V	I	G	I	V	V	M
**Pak|NIH|19429573|2024**	V	I	G	I	V	V	M
(*) represents the same amino acid in the reference genome. (-) represents the sequence is partial and the region is not present.							

**Table 3:** Amino acid variation in the non-structural genes of CHKIV study isolates compared with the reference strain and previously reported sequences from Pakistan.

Proteins	NSP1			NSP2			NSP3			NSP4		
Positions	167	376	476	39	54	702	162	373	428	27	52	403
**Thail|MN630017.1| 2019**	I	M	P	Y	D	A	I	T	V	G	P	I
**PakWIV|EPI_ISL_17455568|2017**	*	*	Q	*	*	D	*	*	*	*	*	*
**Pak|AWX90874.1|2018**	*	*	Q	*	*	D	*	*	*	*	*	*
**Pak|MZ726804.1|Nsp1|2016**	*	*	Q	*	*	D	–	–	–	–	–	–
**Pak|MZ726811.1|Nsp1||2017**	*	*	Q	*	*	D	–	–	–	–	–	–
**Pak|UYI58197.1|Nsp1||2022**	*	*	Q	*	*	D	–	–	–	–	–	–
**Pak|ASY01956.1|2017**	*	*	Q	*	*	D	*	*	*	–	–	–
**Pak|UYI58191.1|2022**	*	*	Q	*	*	D	*	*	*	–	–	–
**Pak|MZ701738.1|2022**	*	*	*	*	*	D	*	*	*	–	–	–
**Pak|UYI58188.1|2022**	*	*	Q	*	*	*	*	*	*	–	–	–
**Pak|MZ701718.1|Nsp3|2016**	–	–	–	–	–	–	*	*	*	–	–	–
**Pak|MZ701726.1 |Nsp3||2017**	–	–	–	–	–	–	*	*	*	–	–	–
**Pak|MZ701720.1|Nsp3|2018**	–	–	–	–	–	–	*	*	*	–	–	–
**Pak|MZ726823.1|Nsp4|2016**	–	–	–	–	–	–	–	–	–	*	*	*
**Pak|MZ726821.1 |Nsp4||2017**	–	–	–	–	–	–	–	–	–	*	*	*
**Pak|UYI58204.1|Nsp4|2022**	–	–	–	–	–	–	–	–	–	*	*	*
**Pak|UYI58207.1|Nsp4|2022**	–	–	–	–	–	–	–	–	–	*	*	*
**Pak|UYI58198.1|Nsp4|2022**	–	–	–	–	–	–	–	–	–	*	*	*
**Pak|NIH|19429568|2024**	V	T	*	H	E	D	V	I	A	R	S	V
**Pak|NIH|19429569|2024**	V	T	*	H	E	D	V	I	A	R	S	V
**Pak|NIH|19429570|2024**	V	T	*	H	E	D	V	I	A	R	S	V
**Pak|NIH|19429571|2024**	V	T	*	H	E	D	V	I	A	R	S	V
**Pak|NIH|19429572|2024**	V	T	*	H	E	D	V	I	A	R	S	V
**Pak|NIH|19429573|2024**	V	T	*	H	E	D	V	I	A	R	S	V
(*) represents the same amino acid in the reference genome. (-) represents the sequence is partial and the region is not present												

## Discussion

In September 2024, a localized outbreak of Chikungunya virus (CHIKV) was reported in Chakal Darband, a village in the Mansehra district of KP, Pakistan. This outbreak, which began with an index case—a 38-year-old individual who had recently returned from Gujranwala, Punjab—spread rapidly within the community, affecting multiple households. Entomological surveys confirmed the presence of *Aedes mosquito* larvae, the primary vector for CHIKV transmission, in both indoor and outdoor environments. This outbreak marked the first significant resurgence of CHIKV in the region since the 2016–2017 epidemic, which had resulted in 550 confirmed cases across Mansehra.

Despite the recurring threat of CHIKV in Pakistan, genomic data on circulating strains remain scarce, limiting our understanding of the virus’s transmission dynamics, evolutionary patterns, and potential adaptations to local vectors. This study aimed to address this gap by employing metagenomic next-generation sequencing (mNGS) to investigate the genomic diversity and phylogenetic characteristics of CHIKV during the 2024 outbreak in Mansehra.

Genotyping of the sequenced samples revealed the ECSA genotype, which has been a dominant driver of CHIKV outbreaks globally since 2004, impacting regions across the Indian Ocean, Asia, the Pacific, and the Americas [39]. This genotype has been associated with significant outbreaks in India since 2006 [40], in Brazil from 2015 onward [41,42], and Karachi, Pakistan, during the 2016–2017 outbreak, where partial sequencing data provided initial insights [28,30,43–45]. Our study confirms the circulation of the ECSA genotype in the Mansehra district during the 2024 outbreak, reflecting regional epidemiological dynamics.

Phylogenetic analysis showed high homology (98.91–99.00%) with sequences from the 2016–2017 outbreaks, reinforcing the continuity of the circulating ECSA genotype with little divergence in Pakistan [43,46]. Additionally, the clustering of these study sequences with those from neighboring countries, India and China, as well as from Japan and Kenya, points to a regional correlation, likely influenced by travel and favorable ecological conditions for *Aedes* mosquito vectors [47,48]. The close genetic relationship, particularly with Indian strains, underscores the ongoing epidemiological linkage between Pakistan and its neighboring countries. These findings emphasize the importance of coordinated surveillance and vector control strategies across borders, as trade and travel have historically contributed to the spread of CHIKV during previous outbreaks [47,49].

The study identified several unique mutations in both structural and non-structural proteins of the CHIKV genome. In the structural proteins, mutations were observed in the envelope proteins E1 (I28V, V290I), E2 (Y39H, D54E), and E3 (T16M), which play critical roles in immune evasion, cell entry, and viral attachment. These mutations may influence the virus’s ability to evade host immune responses or enhance its infectivity [50]. In the non-structural proteins, eleven unique mutations were identified in NSP1, NSP2, NSP3, and NSP4, which are involved in viral replication and host immune modulation [51]. For instance, mutations in NSP1 (I167V, M376T) could affect RNA capping and immune evasion [52], while mutations in NSP4 (G27R, P52S, I403V) may alter RNA polymerase activity, potentially impacting viral replication efficiency [53,54].

Notably, none of the sequenced CHIKV genomes carried the E1-A226V mutation, which is associated with enhanced transmission by *Aedes albopictus*. This finding suggests that the 2024 outbreak was primarily driven by *Aedes aegypti*, consistent with previous reports of *Aedes aegypti* dominance in the region [55,56]. However, the absence of this mutation does not preclude the possibility of future adaptations to Aedes albopictus, underscoring the need for ongoing genomic surveillance to monitor emerging variants and their potential impact on transmission dynamics.

The utility of mNGS in this study extended beyond CHIKV sequencing, enabling the detection of a co-infection with GB Virus-C (GBV-C) in one patient. This highlights the potential of mNGS to identify co-circulating pathogens and provide a more comprehensive understanding of disease etiology during outbreaks. However, the high cost and resource requirements of mNGS pose significant challenges for its widespread adoption in resource-limited settings like Pakistan. Currently, the NIH is the only facility in Pakistan equipped with mNGS capabilities, and delays in sample transport and processing often hinder timely outbreak responses. Decentralizing mNGS capacity to provincial laboratories, coupled with investments in infrastructure and training, will be essential for improving genomic surveillance and outbreak preparedness in the future.

This study has limitations. The small sample size of six near-complete genomes, limits the ability to capture the full genetic diversity of circulating strains and may not be fully representative of the outbreak in Mansehra.The study focuses on a single geographical location, limiting broader geographic and temporal analysis. While metagenomic sequencing identified co-infections, their clinical impact was not assessed. The absence of vector surveillance data hinders understanding of transmission dynamics, and sequencing biases may have influenced genome completeness and mutation detection. Finally, the high cost and logistical requirements of mNGS currently limit its widespread use in Pakistan. Larger genomic datasets and integrated epidemiological studies are needed for a comprehensive understanding of CHIKV evolution and spread.

In conclusion, this study provides critical insights into the genomic landscape of CHIKV during the 2024 outbreak in Mansehra, Pakistan. The identification of the ECSA genotype, along with unique mutations in structural and non-structural proteins, suggests ongoing viral evolution and potential adaptations to local vectors. These findings underscore the importance of enhanced genomic surveillance, vector control, and diagnostic capabilities to mitigate future CHIKV outbreaks. Regional collaboration and coordinated public health responses will be essential to address the evolving threat of CHIKV in South Asia.
